# Electrospun Core (HPMC–Acetaminophen)–Shell (PVP–Sucralose) Nanohybrids for Rapid Drug Delivery

**DOI:** 10.3390/gels8060357

**Published:** 2022-06-07

**Authors:** Xinkuan Liu, Mingxin Zhang, Wenliang Song, Yu Zhang, Deng-Guang Yu, Yanbo Liu

**Affiliations:** 1 School of Materials & Chemistry, University of Shanghai for Science and Technology, Shanghai 200093, China; xinkuanliu@usst.edu.cn (X.L.); 203613006@st.usst.edu.cn (M.Z.); wenliang@usst.edu.cn (W.S.); 2 School of Pharmacy, Shanghai University of Medicine & Health Sciences, Shanghai 201318, China; zhangy_21@sumhs.edu.cn; 3 School of Textile Science and Engineering, Wuhan Textile University, Wuhan 430200, China

**Keywords:** HPMC, coaxial electrospinning, core–shell nanohybrids, orodispersible drug delivery, fast dissolution, poorly water-soluble drug

## Abstract

The gels of cellulose and its derivatives have a broad and deep application in pharmaceutics; however, limited attention has been paid to the influences of other additives on the gelation processes and their functional performances. In this study, a new type of electrospun core–shell nanohybrid was fabricated using modified, coaxial electrospinning which contained composites of hydroxypropyl methyl cellulose (HPMC) and acetaminophen (AAP) in the core sections and composites of PVP and sucralose in the shell sections. A series of characterizations demonstrated that the core–shell hybrids had linear morphology with clear core–shell nanostructures, and AAP and sucralose distributed in the core and shell section in an amorphous state separately due to favorable secondary interactions such as hydrogen bonding. Compared with the electrospun HPMC–AAP nanocomposites from single-fluid electrospinning of the core fluid, the core–shell nanohybrids were able to promote the water absorbance and HMPC gelation formation processes, which, in turn, ensured a faster release of AAP for potential orodispersible drug delivery applications. The mechanisms of the drug released from these nanofibers were demonstrated to be a combination of erosion and diffusion mechanisms. The presented protocols pave a way to adjust the properties of electrospun, cellulose-based, fibrous gels for better functional applications.

## 1. Introduction

Cellulose, as one of the most abundant natural resources, has been exploited in the field of drug delivery for many years [1,2,3,4,5,6]. Particularly, it has a wide variety of derivatives, which have different chemical and physical properties for different drug controlled-release performances [7,8,9,10,11]. For example, acetate cellulose, an insoluble derivative, is frequently utilized to provide a sustained drug release profile [12,13,14]. In sharp contrast, HPMC, as a water-soluble drug, is a popular drug carrier for ensuring the fast dissolution of poorly water-soluble drugs [15,16,17,18]. Meanwhile, these derivatives can be combined with other synthetic polymers to offer multiple-phase drug controlled-release profiles, such as the typical double-phase release that includes an initial, immediate release for easing patient symptoms and a later, sustained release for the sake of reduced administration time [19].

However, cellulose’s functional applications have not yet been fully expanded [20,21,22,23,24,25,26]. On the one hand, advanced techniques in science and engineering have been able to improve the functional performances of cellulose and its derivatives [27,28,29,30,31,32,33]. On the other hand, most cellulose derivatives are insert materials, where the active ingredients and other additives need to be encapsulated into the cellulose matrices to gain advantages such as biocompatibility, stability and ease of use [34,35].

Electrospinning, as an electrohydrodynamic technique, is broadly exploited to treat cellulose and its derivatives for biomedical and other applications [36,37,38]. There are numerous research publications and many review articles about this topic [39,40]. The popularity of this interdisciplinary field is due to the usefulness of cellulose derivatives and the ease of the preparation of nanofibers using electrospinning [41,42,43,44,45,46]. A single-step and straightforward operation of electrospinning on a co-dissolving solution of a cellulose derivative and a drug can bring out medicated nanoproducts with unique properties for targeted applications, which have been demonstrated in many investigations [47,48,49,50,51]. What is more, the fast developments of electrospinning in the following three directions have further produced more and more cellulose-based, medicated products in the forms of composites or hybrids. These three directions are: (1) the double-fluid coaxial, side-by-side and tri-fluid complex processes, by which core–shell, Janus and their combined nanostructures of cellulose can be easily prepared directly and robustly [52,53,54,55]; (2) approaching nanofibers on a large scale using the non-needle processes or multiple-needle processes [56,57]; (3) the reasonable combinations of electrospinning and other traditional pharmaceutical methods and, also, the combinations of cellulose derivatives and other excipients [58,59,60,61].

In this study, with a common cellulose derivative, i.e., hydroxypropyl methyl cellulose (HPMC) as the filament-forming matrix and also the drug carrier, its homogeneous electrospun nanocomposite (containing APP from a single-fluid blending process) and its heterogeneous core–shell nanohybrids (having an additional PVP–sucralose shell from a modified, coaxial process) were fabricated and characterized in parallel. A well-known poorly water-soluble drug, acetaminophen (AAP), was explored as the model’s active ingredient [62,63]. The results show that an additional PVP–sucralose shell effectively promotes the fast gelation of HMPC and also the dissolution of AAP, as well as masking the poor taste of AAP for potential oral administration.

## 2. Results and Discussion

### 2.1. The Two Different Working Processes from the Same Apparatus

**Figure 1:**
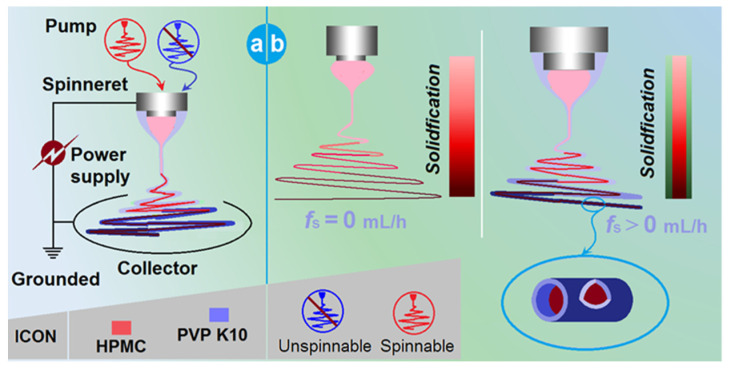
A diagram showing the coaxial apparatus that was explored for both a single-fluid blending process and a modified, coaxial process: (**a**) the parts of the electrospinning apparatus; (**b**) the two processes with different sheath fluid flow rates (*f*_s_).

In an electrospinning system, the spinneret is the most vital component [64,65,66]. This is not only because the electrohydrodynamic process happens around the working fluids and high voltage convergent here, but also because its macrostructures directly determine the nanostructures of the final products and, often, the categories of the electrospinning processes. Figure 1a is a diagram of the elements of a typical, coaxial electrospinning apparatus. The power supply is utilized to provide the electrical energy, the two pumps are exploited to drive the shell and core working fluids quantitatively, the collector is used to collect nanofibers and the spinneret is used to guide the two working fluids in the electrical field in an organized manner. For safety, all the elements must be grounded.

**Figure 2:**
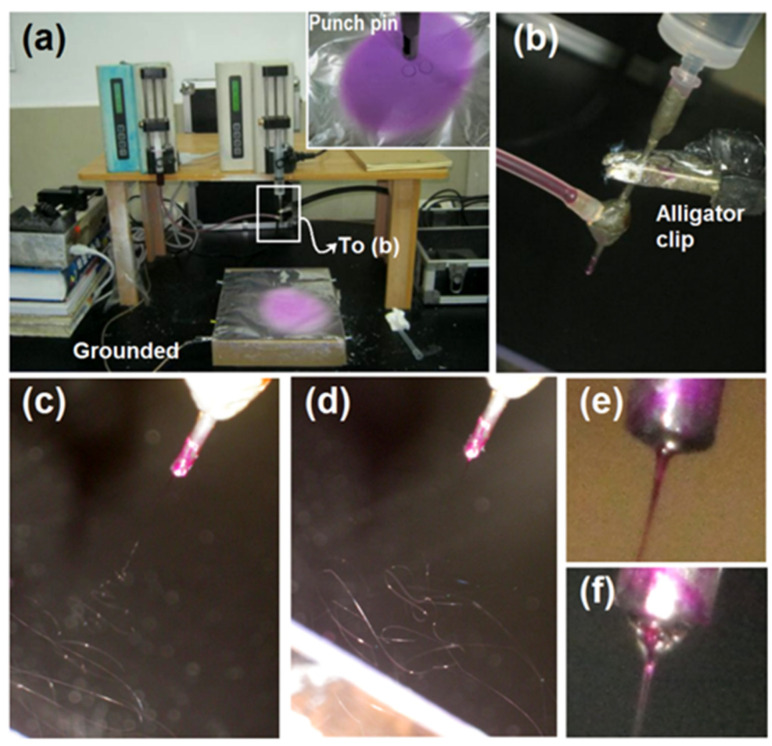
Digital photos exhibiting the preparation processes: (**a**) a whole image of the electrospinning apparatus, the upper-right inset showing the collected nanofibers films prepared by the single-fluid blending process of the core fluid; (**b**) the connection of the spinneret with the two working fluids and the power supply; (**c**) a typical process for creating HPMC-based nanocomposites solely from the core fluid; (**d**) a typical process for producing the core–shell hybrids from the modified coaxial processes; (**e**,**f**) Taylor cones for preparing monolithic composites and core–shell nanohybrids, respectively.

In the traditional sense, the concentric spinneret is utilized to prepare the core–shell nanofibers [67,68]. However, in the coaxial system, when the shell fluid flow rate (*f*_s_) is adjusted to 0 mL/h, the process is degraded to a single-fluid blending process of the core fluid, and the final products are, correspondingly, homogeneous nanocomposites. In contrast, when *f*_s_ is larger than 0 mL/h, the final products are core–shell nanohybrids (Figure 1b). The real implementations of the electrospinning processes are presented in Figure 2. Figure 2a is a whole view of the coaxial electrospinning apparatus which was used for preparing the nanohybrids F2. The collected film showed a slightly pink color. In contrast, the film in the upper-right corner showed a purplish red. Figure 2b shows that the spinneret was easy to connect with two pumps, one by a syringe containing the shell fluid and the other by a highly elastic silicon tubing. An alligator clip was used to transfer the electrical energy to the working fluids. Figure 2c–f shows digital photos of the typical working processes. Figure 2c,d shows the processes of single-fluid and coaxial blending, respectively. The Taylor cone was observed using a camera under a magnification of 12×. For the single-fluid electrospinning of the core fluid, the Taylor cone, which showed a purplish red color, was as shown in Figure 2e. The typical compound Taylor cone when the shell fluid was pumped was as shown in Figure 2f; the purplish core fluid was surrounded by transparent shell fluid. Within the compound Taylor cone, the shell and core fluids were clearly separated into their own colors. However, the collected films of core–shell nanofibers still showed a pink color, as in Figure 2a. Although this color was significantly paler than that of nanofibers F1 from the single-fluid of core liquid shown in the upper-right inset of Figure 2a, it gives a hint that some basic fuchsin in the core solutions might escape to the shell section during the fast drying processes of electrospinning. In the present experiments, both the solutes in the core and shell layers dissolved in the other layers. This means that very small interfacial tensions existed between the shell and core working fluids, which benefit a stable and robust continuous preparation of core–shell nanofibers, although a little diffusion may occur during the bending and whipping processes.

### 2.2. The Morphologies and Inner Structure of the Nanofibers

**Figure 3:**
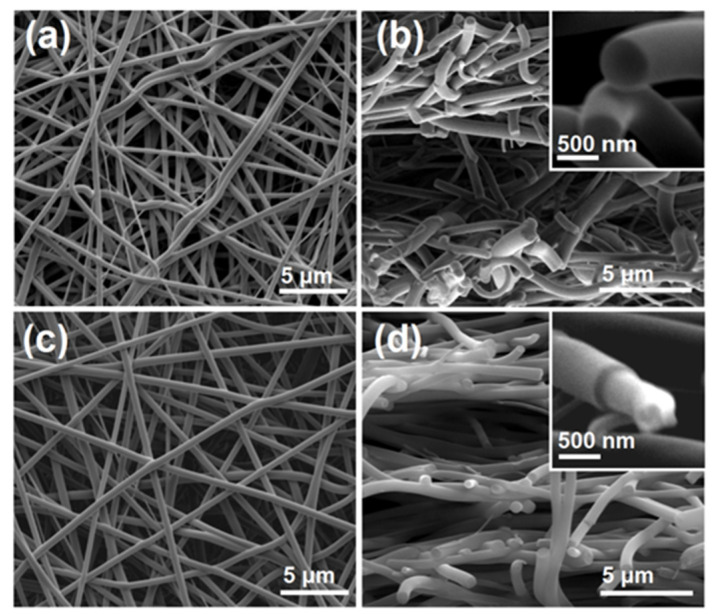
SEM images of the generated nanofibers: (**a**,**b**) the surface and cross-section morphology of the nanocomposites from the single-fluid electrospinning, respectively, the upper-right inset showing an enlarged image for the homogeneous composites; (**c**,**d**) the surface and cross-section morphology of the core–shell nanohybrids from the coaxial electrospinning, respectively, the upper-right inset showing an enlarged image for the heterogeneous hybrids.

The SEM images of the electrospun nanocomposites F1 and the core–shell nanohybrids are included in Figure 3. Figure 3a,b shows the surface and cross-section morphology of the nanocomposites F1 from the single-fluid electrospinning, respectively. It is obvious that these nanofibers were linear without any discerned beads or spindles, suggesting the fine electrospinnability of the core HPMC working fluid [69,70]. These nanofibers had an average diameter of 680 ± 120 nm, which was estimated by calculating the mean values of over 50 points in the SEM images. The upper-right inset of Figure 3b shows an enlarged image of the cross-section of the homogeneous composites F1. No phase separations were observed for either the cross-sections or the surfaces of the nanofibers, giving a hint that the drug AAP presented in the composites F1 in a homogeneous manner with HPMC, most probably on a molecular level.

Figure 3c,d are the SEM images of the surface and cross-section of the core–shell nanohybrids F2 from the coaxial electrospinning, respectively. Although the shell fluid PVP solution had no electrospinnability, straight linear nanofibers could still be robustly prepared. They had an average diameter of 730 ± 80 nm by estimation. The upper-right inset of the figure shows an enlarged image of the heterogeneous hybrids, in which a core–shell structure was observed. This is closely related to the different mechanical properties of the shell and core sections. There were no spindles or beads on the surface of the hybrids or their cross-sections.

**Figure 4:**
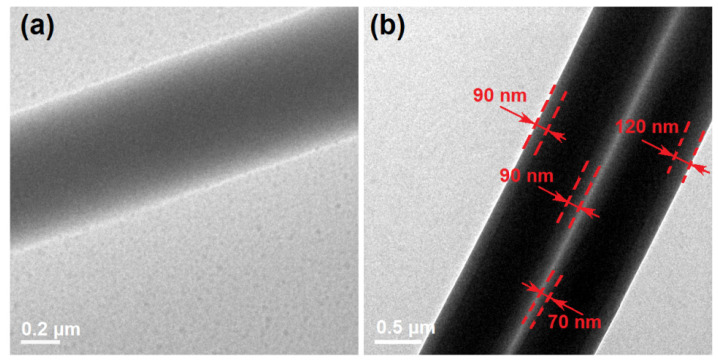
TEM images of the generated nanofibers: (**a**) the nanocomposites from the single-fluid electrospinning; (**b**) the core–shell nanohybrids from the coaxial electrospinning process.

The inner structures of the nanocomposites F1 and the nanohybrids F2 were detected using TEM. The images are exhibited in Figure 4. The nanocomposites F1 showed a gradually reduced gray level from the center of the nanofiber to its boundaries (Figure 4a). It was the thicknesses that made this difference, giving a hint that the different parts of nanofibers F1 had no differences in their elements and density and also no solid phase separation, demonstrating that it was a homogeneous nanocomposite. In sharp contrast, the core–shell hybrids F2 had a significant difference between the core section and the shell section, as shown by the two parallel nanofibers in Figure 4b. By estimation, the core section had a diameter of 540 ± 110 nm and a shell thickness of about 90 nm.

### 2.3. The Physical Forms of Components and Their Compatibility

**Figure 5:**
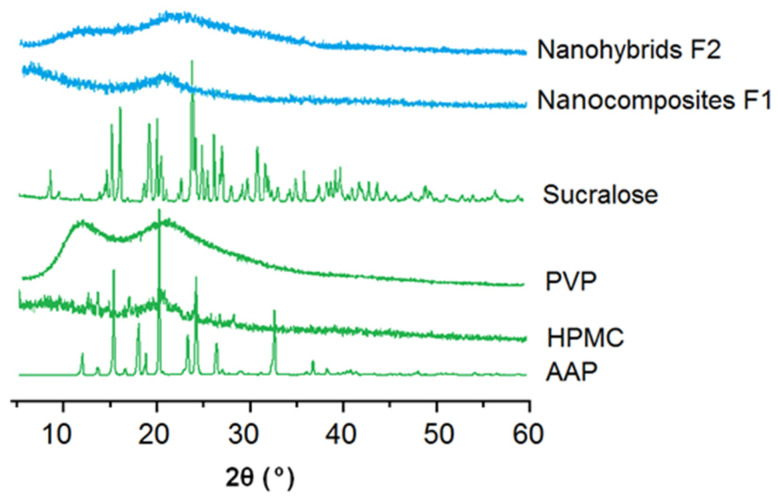
XRD measured results of the starting raw materials (HPMC, ATP, PVP and sucralose) and their different electrospun products.

The measured XRD patterns of the starting raw materials, HPMC, ATP, PVP and sucralose, and their electrospun nanocomposites F1 and core–shell nanohybrids are shown in Figure 5. Just as anticipated, the drug AAP and sucralose had many sharp peaks in their patterns, suggesting that they were crystalline materials originally. HPMC and PVP had no sharp peaks but halos, indicating that they were amorphous polymers. Both the nanocomposites F1 and core–shell nanohybrids had no sharp peaks of the AAP and sucralose, demonstrating that they were converted into amorphous, polymer-based composites. The nanofibers F1 were homogeneous composites containing HMPC and AAP. The core and shell sections of the core–shell nanofibers F2 were composed of homogeneous core composites of HMPC and AAP and a homogeneous shell composite of PVP and sucralose, in general, a hybrid of the core section and shell section.

**Figure 6:**
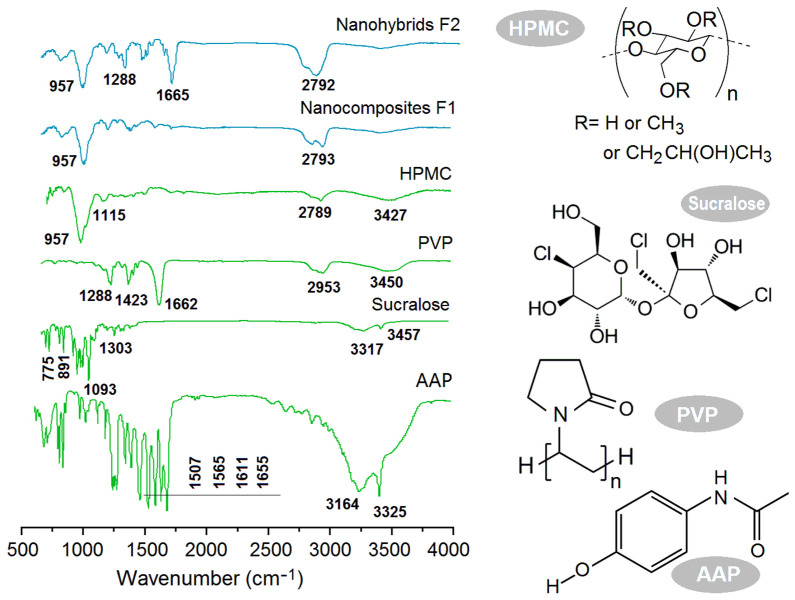
ATR-FTIR spectra of the starting raw materials (HPMC, ATP, PVP and sucralose), their different electrospun products and their molecular formulae.

The ATR-FTIR spectra of the starting raw materials (HPMC, ATP, PVP and sucralose) and their different electrospun products are included in Figure 6. The comparisons of sucralose and AAP spectra with their composites F1 and nanohybrids F2 showed that almost all the sharp peaks in the spectra of the raw materials greatly decreased their intensities or totally disappeared from the spectra of the electrospun products. For example, the characteristic peaks at 1655 cm^−1^ for –C=O and at 1611, 1565 and 1507 cm^−1^ for the benzene ring in the spectra of AAP could not be discerned in the spectra of either the nanocomposites F1 or nanohybrids F2. This showed the close relationship between the secondary interactions of the host polymeric carrier and the guest active ingredient. As shown by their molecular formulae, a sucralose molecule has five –OH groups, whereas, a PVP molecule has many –C=O groups; thus, hydrogen bonding is easy between them and favors the stability of the composites in the shell sections. Similarly, an HPMC molecule has many –OH group, whereas an AAP molecule has a –C=O group, suggesting that the possible hydrogen bonding between them could benefit the stability of nanocomposites F1 and the core section of the core–shell nanohybrids. However, the spectra of nanohybrids F2 had sharp peaks of 1655 cm^−1^ and 957 cm^−1^, which are characteristic peaks of PVP and HPMC. This phenomenon clearly suggests that PVP and HPMC were organized in the core–shell nanofibers in a separate manner, with each having their own region, i.e., a typical hybrid material.

### 2.4. The Hydrophilic Properties

Both HPMC and PVP are water-soluble, polymeric excipients broadly utilized in the pharmaceutical industry [71,72]. However, their dissolution behaviors have differences. PVP is very hygroscopic and can be dissolved in water all at once and, thus, is reported to promote the dissolution of nearly 200 poorly water-soluble drugs [73,74]. In contrast, HPMC is a hydrophilic polysaccharide, which contains partly O-(2-hydroxypropylated) and partly O-methylated cellulose. HPMC shows adjustable solubility based on the degree of substitution [75,76]. In this study, the HPMC was able to dissolve in water by forming a viscous, colloidal solution.

**Figure 7:**
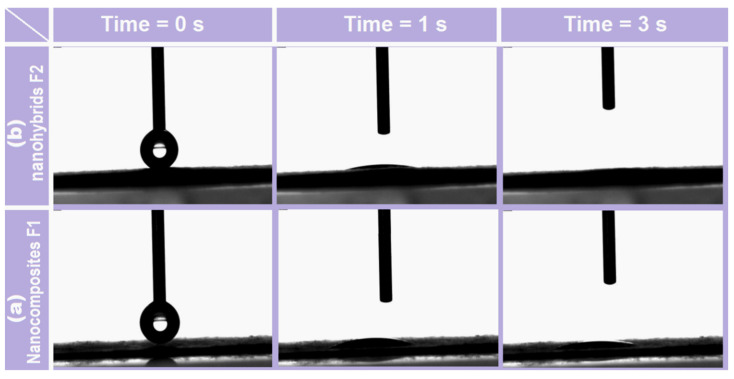
Water contact angle experimental results: (**a**) electrospun core–shell nanohybrids; (**b**) electrospun nanocomposites.

Shown in Figure 7 are the performances of nanocomposites F1 and core–shell nanofibers F2. When a drop of water (3 μL) was placed on their surfaces, the recession processes showed significant differences. In 1 s, the water contact angles for F1 (Figure 7a) and F2 (Figure 7b) were 11 and 6 degrees, respectively. After 3 s, the water droplet totally disappeared from the surface of F2, but the angle for the HPMC–AAP nanocomposites still remained at 8 degrees. The PVP–sucralose shell layer increased the hydrophilicity of the fibrous films.

**Figure 8:**
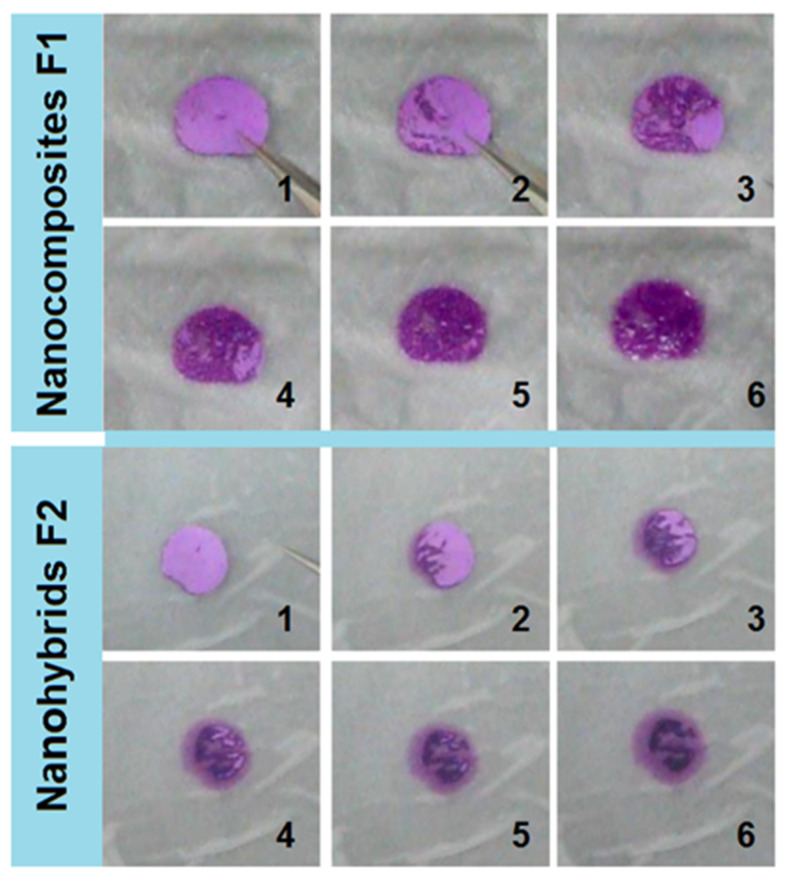
Fast disintegrating experimental results on the wet paper: the electrospun HPMC-based nanocomposites and the electrospun core–shell nanohybrids.

A punch pin with an inner hole of 10 mm, as shown in the upper-right inset of Figure 2a, was utilized to cut circles from the electrospun films. These films were placed on the surface of wet paper (mimicking the tongue). The behaviors of the cut circles caused by the electrospun nanocomposites F1 and core–shell nanohybrids F2 are shown in Figure 8. The nanocomposites F1 showed typical water absorbance and gradual gelation processes. The purplish color was deepened, but the circle showed no significant enlargement. Meanwhile, the purplish color was always within the residues of circle, suggesting that the HPMC gels were able to hold the basic fuchsin well.

The core–shell nanohybrids F2 exhibited different behaviors to nanocomposites F1 in the following aspects: (1) the circle was slightly enlarged, but the pink color was light, which was attributed to the diffusion of basic fuchsin dissolved from the PVP–sucralose shell sections; (2) the colors at different regions were different—some showed a slightly pink color and some showed a deep purplish color, which was closely related to the core–shell nanostructures; (3) the deep purplish color section self-assembled into a strange shape, which was attributed to the movement and aggregation of the core HPMC molecules during the gelation processes and further indicated that the HMPC molecules had a strong capability of holding the basic fuchsin.

### 2.5. The In Vitro Drug Release Profiles and the Mechanisms

**Figure 9:**
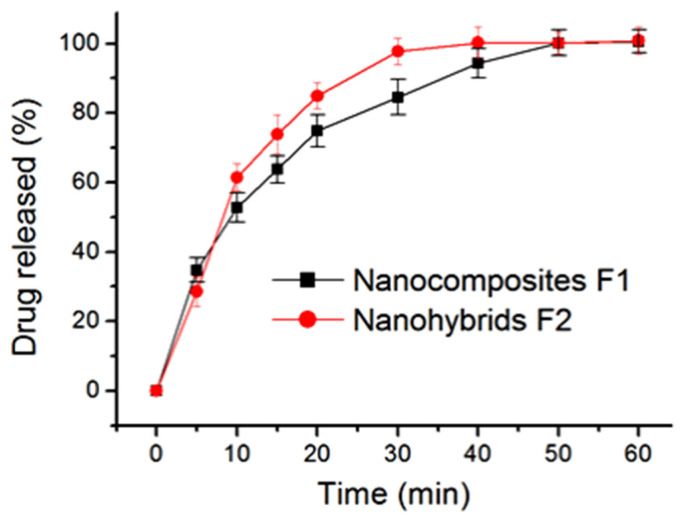
The in vitro dissolution tests of nanocomposites F1 and core–shell nanohybrids.

The in vitro drug release profiles of the electrospun nanocomposites F1 and the core–shell nanohybrids F2 are included in Figure 9. In general, the core–shell nanohybrids F2 were able to provide a faster release effect than the nanocomposites F1. This judgement was made based on the following aspects: (1) after five minutes, nanofibers F1 and F2 released 28.3 ± 4.3% and 34.8 ± 3.5% of the loaded AAP, respectively; and (2) 35.6 and 23.9 min were needed to release 90% of the loaded AAP for nanocomposites F1 and nanohybrids, respectively.

HPMC is frequently utilized both in the pharmaceutical industry and in scientific research as a film coating agent, thickening agent, drug release modifier, drug stabilizer, table binder and suspending ingredient in some liquid dosage forms for oral administration [77,78]. It is well known that the drug release mechanisms of HPMC-based drug delivery systems are often complicated. Various dynamic processes are active during the course of the gelation, diffusion and dissolution processes. These processes are often closely related to the viscosity of HPMC. Higher HPMC often means less erosion and corresponding, longer time period of sustained release. In this study, the drug release data were treated using the famous Peppas equation (Equation (1)) [79]:

(1) $$\begin{array}{c} P=Q_{t}/Q_{0}=k\times t^{n} \end{array}$$

in which Q_t_ and Q_0_ represent the drug released into the dissolution media from its carriers at time point (t), k and n are two constants and P represents accumulative drug release percentage. The drug release mechanisms can be judged by the value of n. It is common knowledge that an n value smaller than 0.45 indicates a diffusion mechanism, a value larger than 0.90 indicates an erosion mechanism and a value between 0.45 and 0.90 represents a complex mechanism involving both diffusion and erosion.

**Figure 10:**
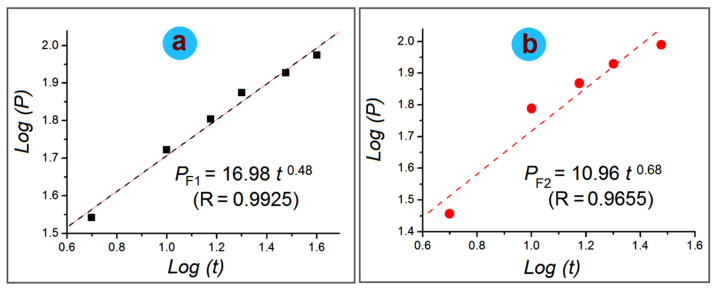
The drug release mechanism judgement: (**a**) the electrospun nanocomposites F1; (**b**) the electrospun core–shell nanohybrids F2.

The regressed equation for the electrospun nanofibers is included in Figure 10. For the electrospun nanocomposites F1, the equation is:

(2) $$\log\left(P\right)=1.23+0.48\log\left(t\right)\mathrm{or}\;P=16.98{\;t}^{0.48}\left(R=0.9925\right)$$

For the electrospun core–shell nanohybrids F2, the equation is:

(3) $$\log\left(P\right)=1.04+0.68\log\left(t\right)\mathrm{or}\;P=10.96{\;t}^{0.68}\left(R=0.9655\right)$$

Thus, it is clear that AAP from the electrospun nanocomposites F1 and the core–shell nanohybrids are all a combination of erosion and diffusion mechanisms because of an n value between 0.45 and 0.90.

### 2.6. The Mechanism of the Influence of Shell PVP on the Gelatin of Core-Medicated HPMC

**Figure 11:**
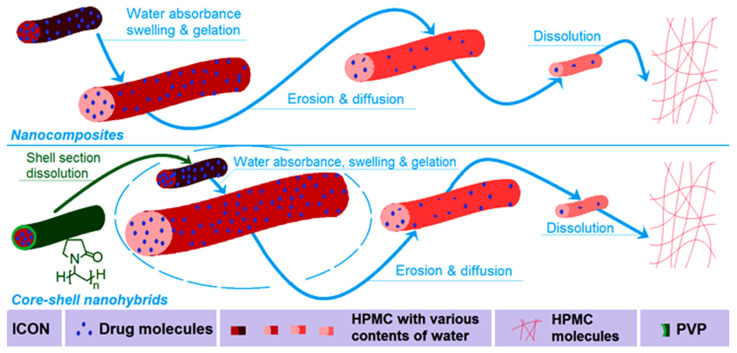
Schematics showing the different behaviors of the monolithic nanocomposites F1 and core–shell nanohybrids F2.

Although the drug release behaviors from the two types of electrospun nanoproduct involved both diffusion and erosion mechanisms, it is obvious that the core–shell nanohybrids F2 were more closed to the erosion mechanism due to a value of 0.68 compared to 0.48 for the nanocomposites F1. A schematic showing the different behaviors of the monolithic nanocomposites F1 and core–shell nanohybrids F2 is given in Figure 11. The replacement of a surface PVP coating in the core–shell nanohybrids F2 adds the benefit of faster initial absorbance of water than monolithic HPMC–AAP nanofibers because of the highly solubility and strong hygroscopicity [73,74]. This case further highlights the easier swelling, gelation and dissolution of HPMC molecules from the core sections. Certainly, the relatively small diameter of the core section in the core–shell nanohybrids F2 compared to the nanocomposites F1 played a role in promoting the faster release of AAP from the nanofibers F2. For potential orodispersible drug delivery, the faster release of the drug, the better it is for the patients. Thus, a shell coating of PVP and sucralose makes the electrospun core–shell hybrids a more welcome product than the electrospun nanocomposites F1. Incidentally, HPMC and PVP are both tasteless and odorless excipients, and, thus, they have broad applications in traditional compressed tablets and medicated films. However, the drug AAP has a bitter taste, which reduces the patients’ compliance. The addition of sucralose with PVP in the shell section endows the core–shell nanohybrids with a favorable taste, and, in turn, could improve the patients’ compliance when exploited as oral, disintegrating films. Using the concept demonstrated here, many new, medicated materials can be further developed through the combination of cellulose-based gels and other types of polymer, e.g., biodegradable PLGA [80]. Particularly, those soluble polymers with a natural source will be able to play a more and more important role in developing new orodispersible drug delivery systems. This is because these polymers often have fine biocompatibility, are non-toxic and have easy processability, and some of them are currently popular in traditional orodispersible tablets [81,82]. Certainly, the coaxial electrospinning, side-by-side electrospinning and also the single-fluid blending processes can be combined with traditional methods (such as casting films) for treating cellulose-based gels to offer sustained release and multiple-phase release profiles [83,84,85].

## 3. Conclusions

With HPMC as a key filament-forming polymeric matrix, both single-fluid blending electrospinning and modified, coaxial electrospinning were implemented to preparing HPMC–AAP monolithic nanocomposites F1 and core (HPMC–AAP)–shell (PVP–sucralose) heterogeneous nanohybrids F2. Both F1 and F2 had linear morphologies without any discerned beads of spindles on them, as demonstrated by the SEM images. TEM images verified that F1 were homogeneous nanocomposites, and F2 were core–shell nanohybrids containing two layers of composites. XRD results suggested that AAP presented in F1 and F2 in an amorphous state. This was attributed to the favorable interactions between APP and HPMC, which were demonstrated by ATR-FTIR measurements. Water contact angle experiments and tongue-mimicking tests clearly demonstrated the differences between F1 and F2 in their hydrophilicity and gelation processes. In vitro dissolution tests demonstrated that the core–shell hybrids F2 were able to offer a faster release of the loaded AAP. The drug release was demonstrated to be controlled by a complex combination mechanism involving both diffusion and erosion. The present study shows a new way of adjusting the properties of electrospun, cellulose-based nanofibers for better drug delivery applications.

## 4. Materials and Methods

### 4.1. Materials

Acetaminophen AAP (white powders, purity 99.8%) was obtained from Hua-Shi Big Pharmacy (Shanghai, China). Hydroxypropyl methyl cellulose (HPMC, white powders, 2910, 5 cps, *M*_n_ = 428,000 g/mol, methoxy content 28.0–30.0%, hydroxypropoxy content 7.5–12.0%) was purchased from Shandong Fine Chemical Co., Ltd. (Jinan, China). Sucralose, polyvinylpyrrolidone (PVP K10, *M*_w_ = 8000) dichloromethane (DCM), anhydrous ethanol and basic fuchsin were bought from Shanghai Chemical Regents Co., Ltd. (Shanghai, China). All other chemicals were analytical reagents. Water was doubly distilled before usage.

### 4.2. Preparations

The electrospinning instrument was homemade and comprised four typical parts: one power supply, two fluid drivers, one collector and one spinneret. After some pre-experiments, the working fluids and experimental conditions were fixed as follows. The core fluid contained 8.0 g HPMC and 2.0 g AAP in a mixture of DCM and anhydrous ethanol with a volume ratio of 1:1. A 10^−3^ mg/mL amount of basic fuchsin was added into the core fluids for optimizing the working processes and also exhibiting the disintegrating measurement. The shell fluid contained 10.0 g PVP K30 and 2.0 g sucralose in anhydrous ethanol.

Two types of electrospun nanofiber were fabricated. One was the monolithic, fibrous nanocomposite (denoted as F1) from single-fluid electrospinning of the core liquid; the other was the core–shell fibrous nanohybrid (denoted as F2) from modified, coaxial electrospinning. The applied voltages were adjusted to ensure continuous spinning and that, meanwhile, no droplets were dropped during the working processes; the values were between 11 and 14 kV. The core fluid flow rate (*f*_c_) was fixed at 2.0 mL/h. The sheath fluid flow rate (*f*_s_) was 0.0 mL/h and 0.5 mL/h for generating F1 and F2, respectively. The ambient conditions were a temperature of 21 ± 4 °C and a relatively humidity of 52 ± 5%.

### 4.3. Characterizations

#### 4.3.1. Morphologies and Inner Structures

The morphologies of electrospun nanofibers were assessed using scanning electron microscope (SEM, Quanta FEG450, FEI Corporation, Hillsboro, OR, USA). The cross-sections of fibers were prepared by manually breaking the fibrous strip after immersion in liquid nitrogen for about 20 min. The inner structures were evaluated using transmission electron microscope (TEM, JEM2200F, JEOL, Tokyo, Japan) under an applied electron voltage of 300 kV.

#### 4.3.2. Physical Forms and Compatibility

All the raw materials (HPMC, PVP, sucralose and AAP) and their electrospun products F1 and F2 experienced X-ray diffraction (XRD, Bruker-AXS, Karlsruhe, Germany) tests. Attenuated total reflectance Fourier-transform infrared (ATR-FTIR, PerkinElmer, Billerica, MA, USA) was exploited to investigate the compatibility between the polymeric carriers and active ingredients.

#### 4.3.3. Properties

Water contact angle measurements and a homemade experiment on wet paper were conducted to evaluate the fast disintegrating properties of the electrospun nanocomposites F1 and core–shell nanohybrids F2, and the gelation processes were recorded using a camera (PowerShot SX50 HS, Canon, Tokyo, Japan).

#### 4.3.4. Functional Performances

In vitro dissolution tests were carried out using paddle methods according to the Chinese Pharmacopoeia (Ed. 2020). An RCZ-8A dissolution apparatus (Tianjin University Radio Factory, Tianjin, China) was used. Samples equivalent to 20 mg AAP were placed into the dissolution vessels in which 600 mL physiological saline (PS) was kept at a temperature of 37 ± 1 °C. The paddle rotation rate was 50 rpm. A UV–vis spectrophotometer (UV-2102PC, Unico Instrument Co., Ltd., Shanghai, China) was used to measure the AAP concentration at a λ_max_ = 243 nm. All the measurements were repeated 6 times.

## Data Availability

The data supporting the findings of this manuscript are available from the corresponding authors upon reasonable request.
